# The pronounced cytotoxic effects of chimeric antigen receptor T cells targeting B7-H3 in organoids and liver xenografts derived from colorectal cancer patients

**DOI:** 10.1038/s41416-025-03114-1

**Published:** 2025-07-28

**Authors:** Yuling Sheng, Li Yan, Qi Liu, Yifan Peng, Jingyun Tan, Wenhua Li, Wei Mao, Wenqing Wei, Yanyun Chang, Linlin Cao, Yi Tan, Yanlin Xiao, Wenyong Zhang, Jing Gao, Yang Xu, Changzheng Du

**Affiliations:** 1 Key University Laboratory of Metabolism and Health of Guangdong, Department of Biochemistry, School of Medicine, Southern University of Science and Technology, Shenzhen, Guangdong PR China; 2 Department of Human Cell Biology and Genetics, School of Medicine, Southern University of Science and Technology, Shenzhen, Guangdong PR China; 3 Department of Unit III & Ostomy Service, Gastrointestinal Cancer Center, Beijing Cancer Hospital & Institute, Beijing, PR China; 4 Department of Oncology, Peking University Shenzhen Hospital, Shenzhen, Guangdong PR China; 5 Cancer Center, Beijing Tsinghua Changgung Hospital, School of Clinical Medicine, Tsinghua Medicine, Tsinghua University, Beijing, PR China; 6 Peking University People’s Hospital, Beijing, PR China; 7 School of Medicine, Southern University of Science and Technology, Shenzhen, Guangdong PR China

**Keywords:** Colorectal cancer, Tumour immunology

## Abstract

**Background:**

The application of chimeric antigen receptor (CAR)-T cells in solid tumors is hindered due to the lack of specific tumor antigen and limited clinical efficacy. Our aim is to develop and validate novel CAR-T cell therapy against metastatic colorectal cancer (CRC).

**Methods:**

By analyzing the expression of B7-H3 in CRC tissue and cell lines using immunohistochemistry (IHC) and flow cytometry, respectively, we identified B7-H3 as a potential target in CRC. We thereby developed CAR-T cells targeting B7-H3 (B7-H3 CAR-T) and evaluated their anti-tumor activity in vitro and in vivo, using patient-derived organoids (PDOs) and xenograft (PDX) models to validate its translational potential.

**Results:**

In our cohort of 170 CRC patients, B7-H3 was significantly upregulated in CRC tumors compared to paratumor tissue, as determined by IHC staining. When co-cultured with CRC cells or PDOs, B7-H3 CAR-T cells exhibited a dose-dependent cytotoxicity in vitro. Furthermore, B7-H3 CAR-T cells effectively controlled tumor growth and metastasis in vivo, significantly prolonging survival time for the tumor-burden mice through cytotoxic killing and potential immune regulatory effects, demonstrated in both CRC cell-based and PDX-based metastatic models.

**Conclusions:**

These findings underscore the potential efficacy of B7-H3 CAR-T cells for treating metastatic CRC and highlight its translational value.

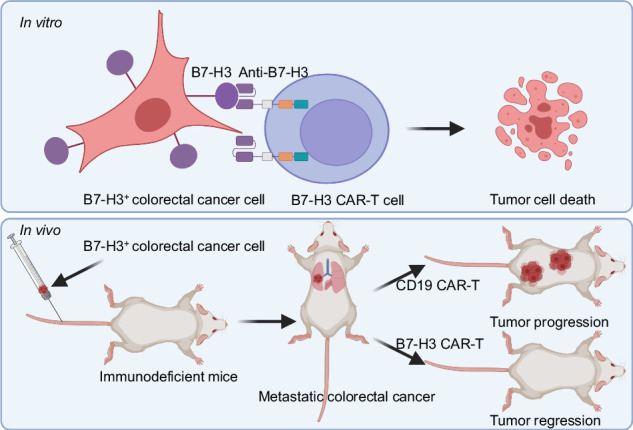

## Introduction

Colorectal cancer (CRC) ranks as the third most common cancer and the second leading cause of cancer-related death globally [1]. Despite the contribution of screening to reducing CRC incidence and mortality, approximately 50% of patients were diagnosed with or developed to incurable distant metastasis after surgery [2, 3]; which remains a formidable challenge for oncologists, notwithstanding that target- and immune-therapy has modestly improved overall survival for the unresectable late-stage disease [4, 5]. There is an urgent requirement to develop more effective treatment strategies for metastatic CRC.

As an innovative treatment, chimeric antigen receptor T (CAR-T) cell therapy towards relapsed or refractory tumors has presented outstanding efficacy in hematologic tumors [6, 7], but its clinical application against solid tumors still faces a number of challenges including difficulties in antigen selection, limited tumor trafficking and infiltration, short persistence of CAR-T cells, and uncertainties regarding clinical safety and tolerability [8]. The most significant challenge that CAR-T cell therapy faces in CRC is the absence of specific antigens suitable for CAR-T cell targeting [9]. Unlike the hematologic malignancies, the heterogeneity of solid tumors always attenuates the killing effect of CAR-T cells by evading antigen-triggered T cell activation [10]. However, recent findings with Claudin18.2-redirected CAR-T cells have shown promising efficacy against gastric cancer in clinical trial [11], providing compelling evidence for the potential applicability of CAR-T cell therapy in treating digestive system cancers, given the identification of an appropriate antigen. Different with gastric cancer, Claudin18.2 is hardly expressed in CRC [12], therefore more feasible tumor-antigens are required for CAR-T cell therapy in CRC.

In this study, we explored the potential of utilizing B7 homolog 3 protein (B7-H3, also known as CD276) as a target for CAR-T cell therapy in CRC, based on our preliminary studies demonstrating a promising safety and efficacy of this treatment in other solid tumors [13]. Our findings revealed a significantly higher expression of B7-H3 in CRC tumors compared to normal tissue, and a favorable killing effect of B7-H3-redirected CAR-T cells against metastatic CRC in vitro and in vivo without obvious toxicity, highlighting the potential of B7-H3-redirected CAR-T cells as a promising therapeutic strategy for metastatic CRC.

## Materials and methods

### Cell lines and cell culture

Human CRC cell lines (HT29, SW620, HCT116, LS174T, LoVo) and HEK293T cells used in this study were obtained from American Type Culture Collection (USA) or Invitrogen (USA). HT29, LS174T and LoVo cells were cultured in RPMI 1640 medium (Gibco, USA). SW620, HCT116 and HEK293T cells were cultured in Dulbecco’s modified Eagle’s medium (DMEM, Gibco, USA). All cell lines were identified by short tandem repeat (STR) profiling and tested mycoplasma-free. These cell lines were maintained in the corresponding medium supplemented with 10% fetal bovine serum (FBS, Gibco, USA), 100 U/mL penicillin and 100 µg/mL streptomycin (Gibco, USA) at 37 °C with 5% CO_2_. All CRC cell lines were transduced with retrovirus encoding the GFP reporter and selected by puromycin (1 μg/ml, MCE, USA). Luciferase expressing HT29 and HCT116 cells were obtained using lentivirus and selected by blasticidin (10 μg/ml, MCE, USA).

### Plasmid construction

To obtain B7-H3 specific CAR cassettes, the plasmid was constructed according to the protocols described previously [13]. Briefly, the DNA fragment encoding B7-H3 specific single-chain variable fragment (scFv) derived from monoclonal antibody (mAb) 376.96 was synthesized and cloned into the γ retroviral vector containing the human CD8a hinge and transmembrane domain, CD28 intracellular costimulatory domain, CD3ζ intracellular signaling domain followed by a mCherry reporter under the IRES bicistronic expression cassette (Fig. S2A). CD19 specific CAR (clone FMC63) was used as control, which was constructed previously [14].

### Virus production and infection

Viruses for cell infection were packaged in HEK293T cells according to the manufacturer’s instruction. For retrovirus production, total 3 × 10^6^ of HEK293T cells were seeded in 10 cm cell culture dish and transfected with the plasmid mixture of the transfer vector, the PegPam plasmid and the RDF plasmid using TransIt-LT1 transfection reagent (Mirus Bio, USA). For lentivirus production, the luciferase vector, pMD2.G plasmid and psPAX2 plasmid transfected to HEK293T cells using Lipofectamine 2000 (Invitrogen, USA). Virus supernatants were collected 48 and 72 h post transfection. After filtration with 0.45 μm filter, the viruses were added to the cells for infection and subsequent screening.

### Generation of B7-H3 specific CAR‑T cells and CAR detection

Human peripheral blood mononuclear cells (PBMCs) were purchased from commercial vendor (Oribiotech, UK) and were stimulated by 1 μg/mL plate-bound CD3 (Miltenyi Biotec, Germany) and CD28 (BD Bioscience, USA) mAbs for 48 h. For retroviral transduction, virus supernatants were added to a retronectin coated plate and centrifuged at 2000 g for 90 mins. After centrifugation, virus supernatants were removed and activated PBMCs were added into each well at 1.25 × 10^5^/mL. On day 3 post transduction, T cells were collected and expanded in T cell medium containing 45% Click’s media (Irvine Scientific, USA), 45% RPMI 1640, 10% FBS with IL-7 (10 ng/mL; Peprotech, USA) and IL-15 (5 ng/mL; Peprotech, USA) as described previously [15]. On day 6 post transduction, the transduction efficiency was detected based on mCherry expression or the surface expression of B7-H3 specific CAR molecule. On day 10–14 post transduction, T cells are harvested for in vitro and in vivo experiments.

### Reverse transcription and quantitative PCR (RT-qPCR)

Total RNA from tumor and paratumor tissue was isolated using the RNA extraction kit (Vazyme, China) following the manufacturer’s protocol. Reverse transcription of total RNA (1 μg) was performed using Reverse Transcriptase Kit (Vazyme, China), followed by Real-time PCR using Green PCR Kit (TransGen, China) on a QuantStudio 7 Flex system (Thermo fisher, USA), with the primers of *B7-H3* (Forward: GAGGAGAATGCAGGAGCTGA, Reverse: TGGTCCTCATGGTCAGGCTA) and *B2M* (Forward: AGGCTATCCAGCGTACTCCA, Reverse: CTGCTTACATGTCTCGATCCCA; as internal control). Fluorescence signal of Real-time PCR was converted to cycle times (CT) normalized to internal control (ΔCT), and the expression level of *B7-H3* was expressed as 2^-ΔCT^.

### Patient materials and Immunohistochemistry (IHC)

Paired tumor and paratumor tissues were collected retrospectively from 170 patients with CRC who underwent surgery in Beijing Cancer Hospital between January 2004 to February 2010 (supplemental Table S1). The inclusion criteria included pathological diagnosed adenocarcinoma, radical surgery and intact follow-up data. The exclusion criteria included familial adenomatous polyposis (FAP) or Lynch syndrome. Each patient signed a written informed consent for the use of their medical information and tissue samples for research.

The specimens were fixed with 4% paraformaldehyde (Sigma-Aldrich, USA) and embedded in paraffin. All the samples were collected in tissue microarray slides. After deparaffination and rehydration, the sectioned slices were subjected to antigen retrieval, endogenous peroxidase inactivation and blocking non-specific antibody binding with 10% normal goat serum (Gibco, USA). The sections were stained with rabbit anti-human B7-H3 primary antibody (Cell Signaling Technology, CST, USA) overnight at 4 °C and then incubated with horseradish peroxidase (HRP)-conjugated goat anti-rabbit secondary antibody (Invitrogen, USA) for 1.5 h at 25 °C, followed by diaminobenzidine (DAB) chromogen (CST, USA) development and counterstained with Hematoxylin (Sigma-Aldrich, USA). Finally, the slices were dehydrated and digitally imaged by Olympus TH4-200 microscope (Japan). Two senior pathologists who were blinded to clinical data assessed the B7-H3 expression using immunoreactive score (IRS) independently. The IRS is determined by multiplying the staining intensity in four gradations (negative, 0; weak, 1; moderate, 2; strong, 3) with the percentage of positive cells in five gradations (<5%, 0; 5–25%, 1; 25–50%, 2; 50–75%, 3; >75%, 4). The samples were classified as “high-level expression” or “low-level expression” based on a cut-off score of 6. All agents and kits used in this study were summarized in Table S2.

### Flow cytometry (FCM) assay

For B7-H3 staining on CRC cells, cell suspensions were washed using PBS before staining with PE-conjugated B7-H3-specific antibody (clone MIH42, Biolegend, USA) at 4 °C for 30 mins. Isotype controls were performed in parallel for each cell line to determine positive staining. The subsets of CAR-T cells were identified by staining with anti-CD4, or anti-CD8 antibodies and subsequently categorizing them as effector T cells (Teff, CD45RA^+^CCR7^-^), effector memory T cells (Tem, CD45RA^-^CCR7^-^), central memory T cells (Tcm, CD45RA^-^CCR7^+^), and stem cell memory T cells (Tscm, CD45RA^+^CCR7^+^). Stained cells were analyzed by using a Novocyte Quanteon flow cytometer (Agilent, USA). The results were analyzed by using FlowJo software (v10.8.2, TreeStar Inc, Ashland, USA).

### Co-culture experiments

We performed co-culture experiment to evaluate the cytotoxicity of CAR-T cells against CRC cells. On day 0, GFP^+^ CRC tumor cells were seeded in 12-well plates at a density of 1 × 10^5^ cells/well and let it settle overnight. On day 1, B7-H3 CAR-T cells were added at an effector-to-target cell (E: T) ratio of 2:1, 1:1 or 1:2. CD19 CAR-T cells at E:T ratio 2:1 were used as negative control. On day 4, all cells were collected and the number of residual tumor cells and CAR-T cells were measured by flow cytometer. CAR-T cells were identified by the expression of mCherry and the residual tumor cells by the expression of GFP. Each experiment was independently replicated four to five times.

### Enzyme-linked immunosorbent assay (ELISA)

Culture supernatant was collected on day 2 of co-culture experiment and ELISA was performed in duplicates using human IFN-γ, IL2 and TNF-α Duoset ELISA kits (R&D Systems, USA), according to the manufacturer’s instructions.

### Patient-derived organoids (PDOs) co-culture assay

PDOs of primary CRC tissue were obtained from Peking University Shenzhen Hospital, and were established and maintained as previously described [16]. Upon reaching a diameter of 100 μm ~ 200 μm, the PDOs were passaged using TrypLE Express enzyme (Gibco, USA). After digestion into single cell suspension, the PDOs were incubated with CellTracker™ Green CMFDA (Invitrogen, USA) at 37 °C for 45 min and then washed twice with PBS. The stained PDOs were suspended in intestinal cancer organoid medium and seeded in a 96-well plate at a density of 10,000 per well. B7-H3 CAR-T and control CAR-T cells were labeled with CytoTrace™ Red CMTPX (AAT Bioquest, USA) and added to the corresponding 96-well plate at E: T of 1:2, 1:1, and 2:1 respectively. The plates were maintained at 37 °C with 5% CO_2_ for 72 h. Imaging was performed using the Operetta® High Content Imaging System (PerkinElmer, USA).

### Xenograft mouse models

Five- to six-week-old NSG mice (female: male =1:1) were purchased from Shanghai Model Organisms Center (Shanghai, China). All mice were maintained in a specific pathogen-free (SPF) facility and acclimated for one week prior to the experiments. All animal procedures were approved by the Institutional Animal Care and Use Committee of Southern University of Science and Technology. For systemic metastatic model, a total of 1.5 × 10^6^ luciferase-expressing HT29 cells or HCT116 cells were injected intravenously into each mouse via the tail vein. Each group included at least five mice per experiment and repeated three times. Tumor growth was monitored weekly by bioluminescence imaging. On day 7 after tumor cell inoculation, mice were randomly assigned to two different groups (coin tossing method) and treated with 5 × 10^6^ CD19 or B7-H3 specific CAR-T cells respectively. Mice without tumor or with unexpected deaths were excluded from analysis. The mice were administered equal doses of CAR-T cells via intravenous and intraperitoneal injection. The body weight was measured twice a week and the survival time of the tumor-bearing mice were recorded. The outcome assessor was blinded to the group allocation throughout the study. The initiation of CAR-T therapy was designated as Day 0, and the termination of observation occurred on Day 49. The mice were euthanized in accordance with the institutional guidelines when they were detected signs of discomfort or when the luciferase signal reached 1 × 10^10^ photons/second (p/s).

### Patient-derived xenograft (PDX) models for CRC liver metastasis

PDX samples were obtained from resected primary tumor tissues provided by Peking University Shenzhen Hospital and the expression of B7-H3 was identified by IHC analysis. Firstly, fresh tissue fragments (2 × 2 × 2 mm^3^/fragment) were implanted subcutaneously into the right flank of NSG mice under sterile conditions. Tumor growth was assessed and calculated twice weekly as follows: V = 0.5 × length × width^2^. Then mice were euthanized and tumor tissues were cut into multiple fragments (2 × 2 × 2 mm^3^/fragment) for subsequent implantation when tumors reached a volume of 250 mm^3^. To establish liver metastasis models, the anesthetized mice were placed in a supine position and laparotomy was performed with mid-line incision after disinfection. One fragment was implanted into the left lobe of the mice liver with a puncture needle and bleeding was stopped by gentle pressure with a sterile cotton swab at the puncture site after removal of the needle and finally the abdominal wall was closed with absorbable sutures. Tumor size was assessed by ultrasound measurement weekly and CAR-T cells was administered via intravenous injection one week after tumor inoculation, mice were sacrificed by inhalation of CO_2_ on Day 30 after treatment. Finally, the metastatic tumor tissue size was measured and photographed. The technicians who measured the tumors were blinded to the mice assignments. All the tumors were fixed in 4% paraformaldehyde and were embedded in paraffin, and the slides were stained by hematoxylin and eosin (H.E., Sigma-Aldrich, USA) for pathologic evaluation.

### RNA sequencing (RNA-seq) assay

To evaluate tumor response to CAR-T cell therapy, 6 mice with PDX liver metastatic tumors were treated with B7-H3 (*n* = 3) or control (*n* = 3) CAR-T cells independently, and their tumors were harvested 2 to 3 weeks after treatment. RNA was extracted from the PDX liver metastatic tumors for the construction of libraries using the NEBNext Ultra II Directional RNA Library Prep Kit for Illumina (New England BioLabs, USA) according to manufacturer’s instructions. The sequencing process was performed on the Illumina NovaSeq 6000 platform (Illumina Inc., USA) with an average of 60 million total reads per sample for RNA-seq. The raw read data underwent quality control and preprocessing by Trim Galore (v0.5.0) and FastQC (v0.11.8) [17], followed by mapping to the human genome through HISAT2 software (v2.2.1) [18]. The ggplot2 package and clustering analysis were utilized to visualize the expression of different expressed genes (DEGs) [19]. The functional enrichment analysis was performed by the clusterProfiler software package 4.0, which incorporates the functional annotations from Kyoto Encyclopedia of Genes and Genomes (KEGG) and Reactome databases [19]. Gene Set Enrichment Analysis (GSEA) 3.0 was used to analyze the signaling pathways enriched based on the DEGs.

### In vivo imaging system (IVIS)

To monitor the tumor growth in mice, in vivo bioluminescent imaging was recorded weekly using the IVIS lumina II in vivo imaging system (PerkinElmer, USA). After anesthesia with isoflurane, XenoLight D-Luciferin potassium salt (PerkinElmer, USA) was intraperitoneally injected into mice as substrate at a dose of 200 mg/kg. The IVIS images were captured 10 min after administration to allow substrate distribution. The gray scale photograph and pseudo-color luminescent images were automatically superimposed to identify the location of any bioluminescence-labeled cells. Optical images were displayed and quantified using Living Image software v4.5.2 (PerkinElmer, USA). Regions of interest (ROI) were drawn to evaluate the relative signal intensity of emission and expressed as p/s.

### Ultrasonography and laboratory tests

Mice were anesthetized with 1.5% isoflurane in oxygen, shaved on abdomen, restrained in a supine position, and underwent abdominal ultrasonography examination. Ultrasound images were captured using a high-frequency VisualSonics Vevo 1100 imaging system (Fujifilm, Japan) with a 30 MHz ultrasound probe as described previously [20]. Tumor growth was assessed and calculated weekly as follows: V = 0.5 × length × width^2^. The liver and renal function of mice following CAR-T cell therapy were evaluated by cobas®8000 c702 automatic biochemical analyser (Roche, Swiss).

### Bioinformatics analysis

The expression of B7-H3 and its correlation with clinicopathologic variables and survival in CRC were assessed using data from The Cancer Genome Atlas (TCGA) database, through the Gene Expression Profiling Interactive Analysis (GEPIA) platform [21] (http://gepia.cancer-pku.cn/).

### Statistical analysis

The data were presented as means ± SD. Differences between two groups were assessed using a two-tailed Student’s t-test. Multiple group comparisons were analyzed by one-way analysis of variance (ANOVA). Survival rate was evaluated using Kaplan-Meier survival analysis and compared using the log-rank test. GraphPad Prism 9 (GraphPad Software, USA) and SPSS 24.0 (IBM SPSS Inc., USA) were utilized for all analyses. A two-sided *P* < 0.05 was considered statistically significant.

## Results

### High expression of B7-H3 in CRC suggests its potential as a tumor antigen

To assess the potential of B7-H3 as a therapeutic target in CRC, we firstly analyzed its transcription level using The Cancer Genome Atlas (TCGA) database and found a significant higher expression of B7-H3 in tumor tissue compared to paratumor tissue in CRC (Fig. S1A), whereas Claudin18.2 showed minimal expression in CRC compared to upper gastrointestinal tract cancers such as gastric and pancreatic cancers (Fig. S1B). The expression of B7-H3 was not associated with TNM stage (Fig. S1C) and disease-free survival (Fig. S1D), but was significantly associated with overall survival (Fig. S1E).

**Fig. 1:**
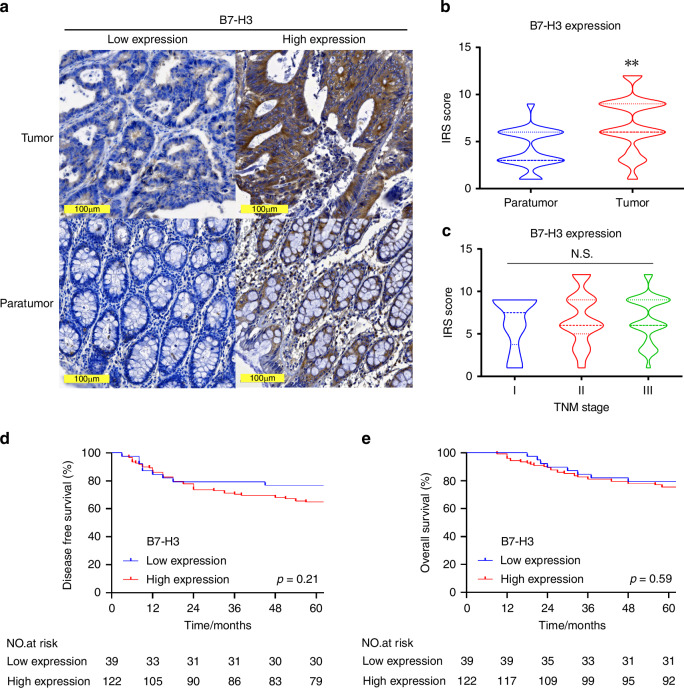
B7-H3 is highly expressed in CRC. **a** Representative images from IHC staining of B7-H3 in tumor and paratumor tissue of CRC. Brown color shows positive staining. Scale bar=100 µm. **b** Expression level of B7-H3 analyzed by IRS score between tumor and paratumor tissue. **c** Expression of B7-H3 in different TNM stages of CRC. **d**, **e** Relationship between B7-H3 expression and disease-free survival (**d**) or overall survival (**e**) analyzed by Kaplan-Meier survival analysis and log-rank test. **p < 0.01; N.S., no significance.

To validate the above findings, a cohort of 170 CRC patients was retrospectively recruited, and the B7-H3 expression was detected in tumor and paired paratumor tissues by IHC (Table S1). The representative slides of B7-H3 staining were shown in Fig. 1a, and a remarkably higher expression of B7-H3 in tumors compared to their matched paratumor tissues was observed (Fig. 1b), which was cross-validated by RT-qPCR using nitrogen-frozen tissues (Fig. S1F–J). In our cohort, no significant association was observed between B7-H3 expression and TNM stage (Fig. 1c), disease-free survival (Fig. 1d) or overall survival (Fig. 1e). All the data above suggested a potential of B7-H3 to be a target of CAR-T cells.

### CRC cells are targeted by B7-H3 CAR-T cells

**Fig. 2:**
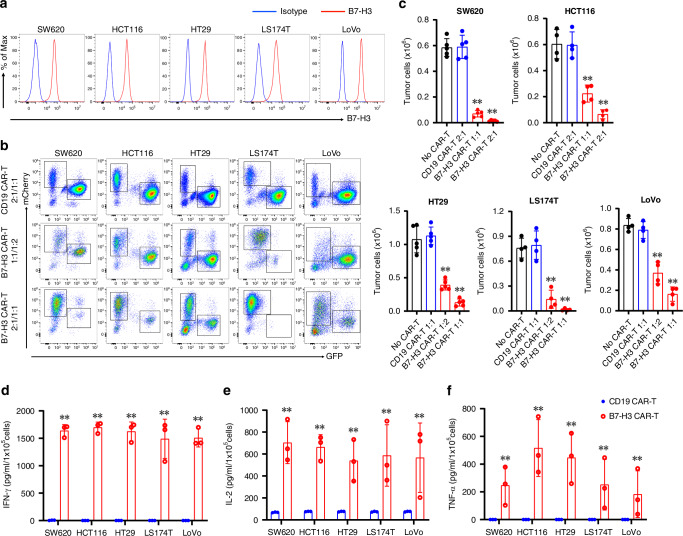
B7-H3 CAR-T cells target CRC in vitro. **a** Representative FCM plots of B7-H3 expression in different CRC cell lines. **b**, **c** CRC cells were co-cultured with CD19 or B7-H3 CAR-T cells for 72 h at an E:T ratio of 1:2, 1:1 or 2:1, respectively; subsequently, the enumeration of living CRC cells was performed using FCM. Representative FCM plots (**b**) and quantification of living tumor cells (**c**) are presented. Data are presented as mean ± SD. **d–f** The quantification of IFN-γ (**d**), IL-2 (**e**) and TNF-α (**f**) released by CAR-T cells in the supernatant of co-culture were measured by ELISA. Data are presented as mean ± SD. **p < 0.01.

To validate the expression of B7-H3 in CRC cell lines, we detected B7-H3 using flow cytometry in five commonly used human CRC cell lines (SW620, HCT116, HT29, LS174T and LoVo), and found a high expression of B7-H3 in all tested cells (Fig. 2a). Thus, the anti-tumor activity of CAR-T cells was assessed against the above five cell lines. We generated a CAR targeting B7-H3 (B7-H3 CAR-T) by using the single-chain variable fragment (scFv) derived from mAb 376.96 which incorporates CD28 endo-domain for co-stimulation (Fig. S2A) [13]. CAR-T cells directed against CD19 (CD19 CAR-T) was utilized as a control. We achieved high (>80%) transduction efficiency in both groups of CAR-T cells in most donors demonstrated by mCherry reporter (Fig. S2B, C). The engineered CAR-T cells were demonstrated with exponential proliferative activity with minimal differences in kinetics between the two groups (Fig. S2D). Subsets of T cells were identified by FCM, indicating no significant difference in the ratios of CD4^+^ T and CD8^+^ T cell between the two groups (Fig. S2E). B7-H3 CAR-T cells showed robust and dose-dependent cytotoxicity towards all 5 CRC cell lines, while the control CD19 CAR-T cells showed minimal tumoricidal activity (Fig. 2b, c). Cytolytic activity of B7-H3 CAR-T cells was accompanied by cytokine release (interferon-γ [IFN‐γ], interleukin-2 [IL-2] and tumor necrosis factor-α [TNF-α]) detected in the culture supernatant (Fig. 2d, e).

### B7-H3 CAR‑T cells are effective in treating metastatic CRC in Xenograft Models

**Fig. 3:**
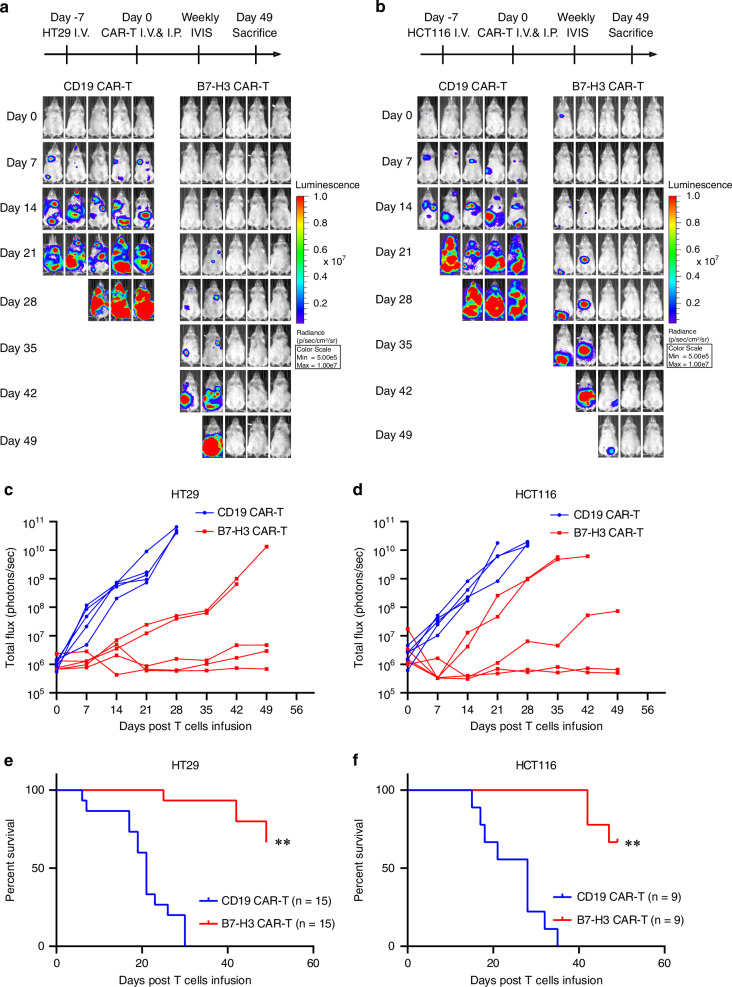
B7-H3 CAR-T cells control metastatic CRC progression in vivo. **a**, **b** Treatment schema and representative bioluminescence images of metastatic CRC tumors in mice bearing HT29 (**a**) and HCT116 (**b**) xenografts. **c**, **d** Bioluminescence kinetics of tumor progression in the metastatic CRC xenografts of HT29 (**c**) and HCT116 (**d**). **e**, **f** Survival curves of mice bearing HT29 (**e**) and HCT116 (**f**) xenografts. Data was analyzed by Kaplan-Meier survival analysis and log-rank test. **p < 0.01.

To validate the safety and anti-tumor efficacy of B7-H3 CAR-T cells against metastatic CRC in vivo, we established xenograft models by intravenously injecting luciferase-expressing CRC cells (HT29 and HCT116). Seven days later, mice were infused with B7-H3 CAR-T or CD19 CAR-T cells via tail vein injection (50%) and intraperitoneal injection (50%), and the metastatic lesions were monitored by IVIS imaging weekly (Fig. 3a, b). B7-H3 CAR-T cells effectively controlled tumor progression in both HT29 and HCT116 derived metastatic xenografts, with 66.67% (10/15) of HT29 and 66.67% (6/9) of HCT116 inoculated mice remained tumor-free at the endpoint of observation. On the contrary, the widely spreading lesions were observed in the mice treated with control CAR-T cells (Fig. 3c, d). Moreover, the mice treated with B7-H3 CAR-T cells displayed a significantly improved survival rate compared to those treated with control CAR-T cells (57% *vs*. 0% in mice inoculated with HT29 cells, *p* < 0.001; 67% *vs*. 0% in mice inoculated with HCT116 cells, *p* < 0.001) (Fig. 3e, f). In addition, no obvious weight loss, liver or renal dysfunction were observed in the B7-H3 CAR-T cells treated mice (Fig. S3). Altogether, the data above suggests that B7-H3 CAR-T cells are effective and safe in treating metastatic CRC in vivo.

### B7-H3 CAR-T cells exhibit superior antitumor activity when targeting PDOs and PDX-based liver metastatic CRC

**Fig. 4:**
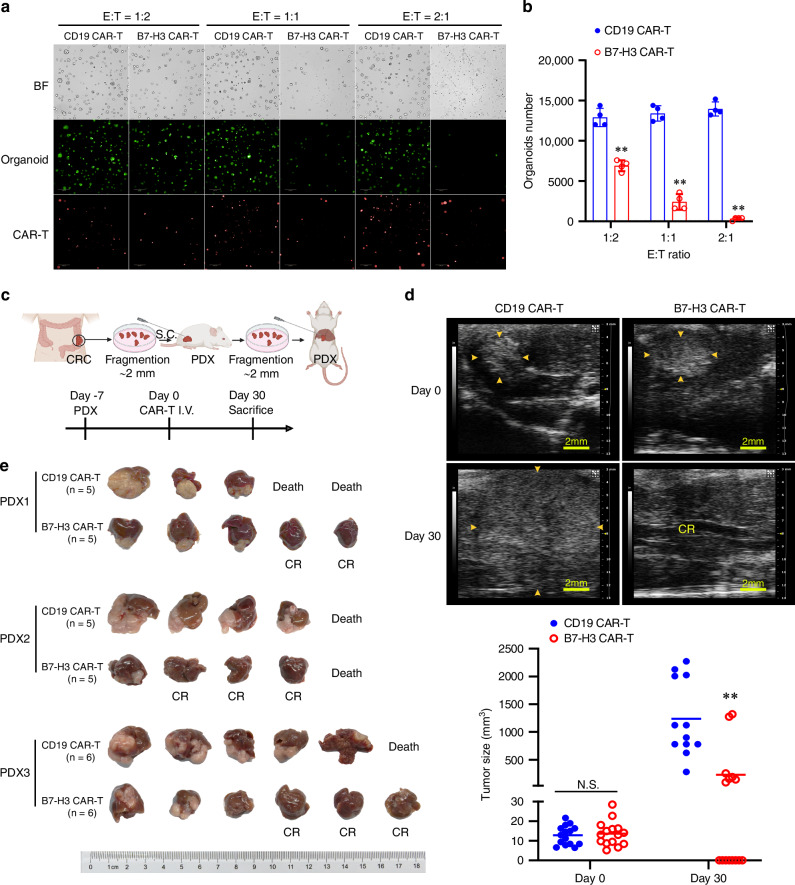
B7-H3 CAR-T cells target patient-derived CRC in intro and in vivo. **a**, **b** Organoids of CRC were co-cultured with CD19 or B7-H3 CAR-T cells for 72 h at an E:T ratio of 1:2, 1:1 or 2:1, respectively; the representative immunofluorescence images (**a**) and quantification of residual living organoids (**b**) are illustrated. BF: bright field; green color, living organoids; red color, CAR-T cells. Scale bar=200 μm. Data are presented as mean ± SD. **c** Schema of the PDX liver metastatic model. **d** Representative ultrasound imaging of the PDX liver metastatic tumors pre- and after treatment with CD19 or B7-H3 CAR-T cells (upper panel), and the statistical results (bottom panel). Yellow triangle arrows show the margin of tumors. Scale bar = 2 mm. CR complete response. Data are presented as mean ± SD. **e** Mice livers and the PDX tumors inside after treatment with CD19 or B7-H3 CAR-T cells. PDX 1–3 represents 3 CRC patient donors. **p < 0.01.

To further explore the translational potential of B7-H3 CAR-T cells in CRC therapy, we assessed their antitumor activity in patient-derived tumor models in vitro and in vivo. Given that PDOs closely resemble CRC tumors in terms of structure and physiological functions [22], we co-cultured B7-H3 CAR-T cells or control CAR-T cells with B7-H3 highly expressed PDOs of CRC to evaluate their antitumor activity (Fig. 4a and Fig. S4A). B7-H3 CAR-T cells exhibited a remarkable cytotoxicity against the PDOs, not only inducing a significant reduction of living organoids (Fig. 4b), but also leading to a remarkable morphological alteration characterized by cell swelling, loss of membrane integrity and loss of structural organization of sphere accompanied by massive cellular debris (Fig. S4B). These findings prompted us to validate their therapeutic efficacy in PDX models in vivo. We thereby established a PDX liver metastasis model using tumor samples (from 3 patients) with high expression of B7-H3 (Fig. 4c and Fig. S4C), treated the mice with B7-H3 CAR-T or control CAR-T cells intravenously (i.v.) via tail vein injection, and monitored the tumor growth by ultrasonography. Our findings revealed that B7-H3 CAR-T cells effectively suppressed liver tumor growth (Fig. 4d, e), with a complete response rate of 50% (8/16). These results underscore the promising potential of B7-H3 CAR-T cells for treating CRC based on their superior antitumor activity against patient-derived tumor models.

**Fig. 5:**
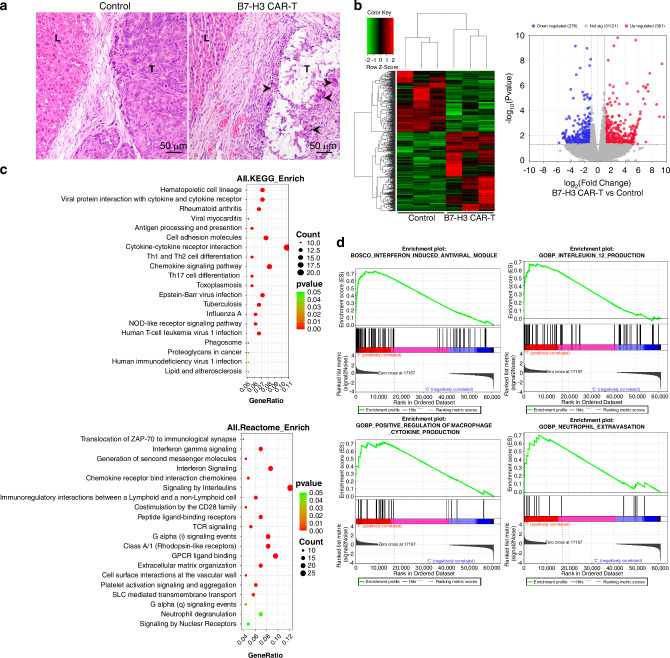
Histological response and gene expression alteration of metastatic CRC following B7-H3 CAR-T cell therapy. **a** Representative sections of PDX liver metastatic CRC tumors treated with control or B7-H3 CAR-T cells (H.E. staining, ×200). L, liver tissue; T, tumor tissue; arrows show multinucleated giant cells surrounding the necrotic tumor tissue. **b** Different expressed genes (DEGs) of tumors treated with control or B7-H3 CAR-T cells, determined by RNA sequencing 2 to 3 weeks after therapy. Left panel, clustered heat map of DEGs; Right panel, volcano plot of all genes, within which DEGs were labelled by red and blue color. **c** Signal pathway enrichment analysis for the DEGs with KEGG (upper panel) or Reactome (Bottom panel) database, linking the DEGs with biological function. **d** Illustration of immune related signal pathways enriched by Gene Set Enrichment Analysis (GSEA).

To gain further insight into the cytotoxic effect of B7-H3 CAR-T cells on the liver metastatic CRC, we assessed the histological response of PDX metastatic tumors to treatment with B7-H3 or control CAR-T cells, as observed in pathological slides (Fig. 5a). Compared to the control group, massive cell death and subsequent cavity-like changes were observed in the B7-H3 CAR-T cell treatment group, accompanied by inflammatory cell infiltration and granulomatous reaction (Fig. 5a). This finding led us to investigate the responses of tumor cells following the targeted attack by CAR-T cells against B7-H3, given that B7-H3 is recognized as an immune checkpoint molecule within tumors [23]. Through RNA sequencing of the treated tumor tissue, we identified a substantial alteration in gene expression profiles, with over 600 different expressed genes (Fig. 5b). Bioinformatic analysis revealed that several immune-related pathways were activated within tumor tissues following therapy with B7-H3 CAR-T cells, including signaling pathways associated with interferons, interleukins, chemokines, T-cell receptors (TCR) and others (Fig. 5c, d and Fig. S5). Notably, the regulation pathways related to macrophage and neutrophil were significantly enriched (Fig. 5d), which aligns with the pathologic findings regarding inflammatory infiltration post-B7-H3 CAR-T cell therapy. Overall, the above findings suggest that CAR-T cells targeting B7-H3 have potential immune regulatory effects on the metastatic CRC tumors.

## Discussion

As an innovative therapeutic strategy, CAR-T therapy has exhibited promising efficacy in treating cancers refractory to conventional therapies [24, 25]. Several ongoing studies are investigating the use of CAR-T cells for metastatic CRC treatment [26, 27], however, one challenge of CAR-T cell therapy in the majority of solid tumors is the absence of cancer-specific antigens suitable for targeting [9]. The identification of target antigens plays a central role in CAR-T cell therapy as it determines the efficacy and safety of the treatment [28, 29]. Unlike gastric cancer in which the Claudin18.2-redirected CAR-T cell therapy has shown a promising therapeutic outcome [11], colorectal cancer is facing a significant challenge due to the need for feasible tumor-antigens suitable for CAR-T cell therapy. Although Claudin18.2 is highly expressed in upper gastrointestinal tract cancers, it is not an appropriate target for CRC given its the low expression rate [30]. Despite numerous molecules being validated in clinical trials, such as CEA, CD133, MUC1, GCC19 and others [31–33], the therapeutic outcomes of CAR-T cells in CRC have not been as favorable as those observed in hematologic malignancies, with the objective response rates ranging from 20% to 40% [31, 33]. Therefore, it is valuable to explore CRC-specific antigens suitable for CAR-T cell treatment. In the present study, to establish the specificity of B7-H3-mediated cytotoxicity, we utilized CD19 CAR-T cells as a control since CD19 is predominantly expressed in B cells but minimally expressed in CRC and other normal tissues. Therefore, using CD19 CAR-T cells allows us to eliminate non-specific immune responses towards tumors and normal organs in immunodeficient animal models.

B7-H3 has been identified as an immune checkpoint that plays a crucial role in inhibiting T cell activation and proliferation, thereby promoting tumor immune evasion [23]. The high expression of B7-H3 in malignant tumors is associated with diminished infiltration of CD8^+^ T cells and suppressed tumor antigen-specific immune responses [34]. Additionally, B7-H3 facilitates tumor migration and invasion, promotes angiogenesis, confers resistance to chemotherapy, and induces endothelium-to-mesenchymal transition through various pathways [35–37]. Therefore, unlike other tumor-associated antigens (TAAs), targeting B7-H3 not only inhibits tumor cells but also enhances immune response in the local microenvironment, thus boosting the anti-tumor immune response [34]. As shown in our study, B7-H3 is particularly suitable for CAR-T cells targeting in CRC due to its nearly 100% positive expression in tumors and the promising anti-tumor activity of B7-H3 CAR-T cells in metastatic CRC. Moreover, targeting B7-H3 with CAR-T cells has the potential to activate immune cells within the microenvironment of metastatic tumors by promoting tumor cells to express immune-activating cytokines or other regulatory factors, as suggested by our findings. Given the current limitations of anti-immune checkpoint therapy in colorectal cancer, which is only applicable to 10–15% of patients with high microsatellite instability (MSI-H) [38], B7-H3 CAR-T cells have the potential to benefit a larger number of patients with incurable distant metastasis, if their efficacy is further confirmed by clinical trials.

In addition to the specificity of tumor-associated antigens, the efficacy of CAR-T therapy is determined by multiple factors, including the engineering of CAR-T cells and patient-related considerations such as administration approach and the tumor immune microenvironment [9, 25]. For instance, peritoneal metastasis is a common form of distant metastasis in CRC and is associated with a short survival time [39]. It has been observed that peritoneal metastasis is resistant to CAR-T cell therapy due to limited infiltration of immune cells [40]. In our study, nearly 80% of mice deaths were attributed to peritoneal metastasis. Drawing inspiration from research on CAR-T cell therapy in gastric cancer [41], we performed intraperitoneal infusion combined with intravenous administration to enhance the distribution of CAR-T cells within the peritoneal cavity [40]. Our results support the local administration of CAR-T cells as an additional enhancement for treating or preventing peritoneal metastasis, by observing an improved control of peritoneal metastasis without severe side effects. To further augment the anti-cancer efficacy of CAR-T cells, it is essential to evaluate T cell activity by biomarkers including PD-1, LAG-3 and TIM-3, which contributes to facilitate the identification of high-quality T cells for clinical application.

Finally, the safety of CAR-T cell therapy is a crucial issue in terms of its potential for clinical application. Although our study and previous reports indicate that B7-H3 CAR-T cells have demonstrated reliable safety in preclinical studies [13], there remain unknown risks in clinical settings due to the significant differences between immune-deficient animal models and real-world patients. One major concern is the potential toxic response resulting from off-target attacks by CAR-T cells. The expression level of B7-H3 is relatively low in most human organs, with notable exceptions including the placenta, prostate, adipose tissue, gallbladder and cervix, suggesting that B7-H3 CAR-T cells may inadvertently target these normal tissues, posing a clinical risk for therapeutic complications, albeit potentially non-lethal, that warrants cautious consideration in clinical practice; particularly for patients with underlying conditions affecting those target organs. Notably, the elevated expression of B7-H3 in placental tissue indicates an exceptionally high risk of B7-H3 CAR-T cell treatment in pregnant women, which should be given considerable attention in clinical trial and practice. In addition, this study did not monitor the dynamic change of the inflammatory cytokines in serum, such as IL-6 and TNF-α; thus, the systemic immune response following B7-H3 CAR-T therapy remains unclear. Despite these limitations, B7-H3 CAR-T cells present a promising therapeutic strategy for patients with metastatic CRC.

## Supplementary information

Supplemental material

## Data Availability

RNA-seq raw data has been deposited to Sequence Read Archive (https://dataview.ncbi.nlm.nih.gov/object/PRJNA1162282?reviewer=is3olcvkqoj2a0q0qlhnoj3tgk for reviewers) under accession number PRJNA1162282. All data in this paper is accessible upon request.
