# Supplementary material for: The pronounced cytotoxic effects of chimeric antigen receptor T cells targeting B7-H3 in organoids and liver xenografts derived from colorectal cancer patients
Source: Br J Cancer. 2025 Jul 28;133(7):1056–65. doi: 10.1038/s41416-025-03114-1 (PMC12480541; doi:10.1038/s41416-025-03114-1)
Supplement: Supplementary file 1 — Supplemental material [file 41416_2025_3114_MOESM1_ESM.docx]

**Table S1. The demographic and clinical material of patients**

| **Clinicopathological parameters** | **No.(%)** |
| --- | --- |
| **Median age**, years (range) | 68 (28-93) |
| **Gender (M:F)** | 85:85 |
| **Tumor location** |  |
| Right side | 78 (45.9) |
| Left side | 92 (54.1) |
| **Histopathological differentiation** |  |
| Well | 15 (8.8) |
| Moderate | 139 (81.8) |
| Poor | 9 (5.3) |
| Mucinous and signet | 7 (4.1) |
| **TNM stage** |  |
| Ⅰ | 13 (7.6) |
| Ⅱ | 63(37.1) |
| Ⅲ | 94 (55.3) |
| **Vascular invasion** |  |
| Yes | 37 (21.8) |
| No | 133 (78.2) |
| **Serum CEA (ng/ml)** |  |
| ≤5 | 80 (47.1) |
| >5 | 81 (47.6) |
| Unknown | 6 (3.5) |
| **Microsatellite status** |  |
| MSI | 26 (15.3) |
| MSS | 144 (84.7) |

MSI, microsatellite instability; MSS, microsatellite stability

**Table S2. Reagents, kits and material information**

| **REAGENT or RESOURCE** | **SOURCE** | **IDENTIFIER** |
| --- | --- | --- |
| **Antibodies** |  |  |
| B7-H3 (D9M2L) XP® Rabbit mAb | CST | Cat#14058 |
| PE anti-human CD276 (B7-H3) Antibody | Biolegend | Cat#351004 |
| PE Mouse IgG1, κ Isotype Ctrl (FC) Antibody | Biolegend | Cat#400114 |
| Brilliant Violet 711™ anti-human CD4 Antibody | Biolegend | Cat#317440 |
| Alexa Fluor® 700 anti-human CD8 Antibody | Biolegend | Cat#344724 |
| PE anti-human CD45RA Antibody | Biolegend | Cat#304108 |
| FITC anti-human CD197 (CCR7) Antibody | Biolegend | Cat#353216 |
| Purified NA/LE Mouse Anti-Human CD28 | BD Bioscience | Cat#555725 |
| CD3 Antibody, anti-human | Miltenyi Biotec | Cat#130093387 |
| Goat anti-Rabbit IgG (H+L) Secondary Antibody, HRP | Invitrogen | Cat#65-6120 |
| **Chemicals and reagents** |  |  |
| DMEM | Gibco | Cat#11995065 |
| RPMI 1640 medium | Gibco | Cat#61870036 |
| Click's Medium | Irvine Scientific | Cat#9195 |
| Colorectal cancer organoid kit | Accurate international biotechnology | Car#M102 |
| Penicillin/streptomycin | Gibco | Cat#15140122 |
| Fetal bovine serum | Gibco | Cat#10099141C |
| PBS | Biosharp | Cat#BL302A |
| Puromycin | MedChemExpress | Cat#HY-K1057 |
| Blasticidin S hydrochloride | MedChemExpress | Cat#HY-103401 |
| TransIT-LT1 Transfection Reagent | Mirus Bio | Cat#MIR2300 |
| Lipofectamine™ 2000 | Invitrogen | Cat#11668030 |
| Recombinant Human IL-7 | Peprotech | Cat#200-07 |
| Recombinant Human IL-15 | Peprotech | Cat#200-15 |
| paraformaldehyde | Sigma-Aldrich | Cat#158127 |
| Goat serum | Gibco | Cat#16210064 |
| Hematoxylin | Sigma-Aldrich | Cat#H3136 |
| 0.25% EDTA-Trypsin | Gibco | Cat#25200072 |
| TrypLE Express enzyme | Gibco | Cat#12605010 |
| Trypan blue stain | Gibco | Cat#15250061 |
| Matrigel Matrix | Corning | Cat#356231 |
| CellTracker™ Green CMFDA | Invitrogen | Cat#C7025 |
| CytoTrace™ Red CMTPX | AAT Bioquest | Cat#22015 |
| XenoLight D-Luciferin potassium salt | PerkinElmer | Cat#122799 |
| TRIzol™ | Invitrogen | Cat#15596026 |
| hematoxylin | Sigma-Aldrich | Cat#H3136 |
| **Cell Lines** |  |  |
| 293T | Invitrogen | Cat#R70007 |
| LoVo | ATCC | Cat#CCL-229 |
| SW620 | ATCC | Cat#CCL-227 |
| LS174T | ATCC | Cat#CL-188 |
| HT29 | ATCC | Cat#HTB-38 |
| HCT116 | ATCC | Cat#CCL-247 |
| **Plasmids** |  |  |
| pLX304 Luciferase-V5 blast | Addgene | Cat#98580 |
| psPAX2 | Addgene | Cat#12260 |
| pMD2.G | Addgene | Cat#12259 |
| B7-H3.CD28.CD3ζ | This paper | N/A |
| CD19.CD28.CD3ζ | This paper | N/A |
| **Critical Commercial Kits** |  |  |
| DAB Substrate Kit | CST | Cat#8059 |
| NEBNext Ultra II Directional RNA Library Prep Kit for Illumina | NEB | Cat#E7760 |
| Human IFN-gamma DuoSet ELISA | R&D Systems | Cat#DY285 |
| Human IL-2 DuoSet ELISA | R&D Systems | Cat#DY202 |
| Human TNF-alpha DuoSet ELISA | R&D Systems | Cat#DY210 |
| FastPure Cell/T issue Total RNA isolution Kit V2 | Vazyme | Cat#RC112-01 |
| HiScript III 1st Strand cDNA Synthesis Kit (+gDNA wiper) | Vazyme | Cat#R312-02 |
| TransStart Top Green qPCR SuperMix | TransGen Biotech | Cat#AQ132-21 |

**Supplemental figures and legends**

**
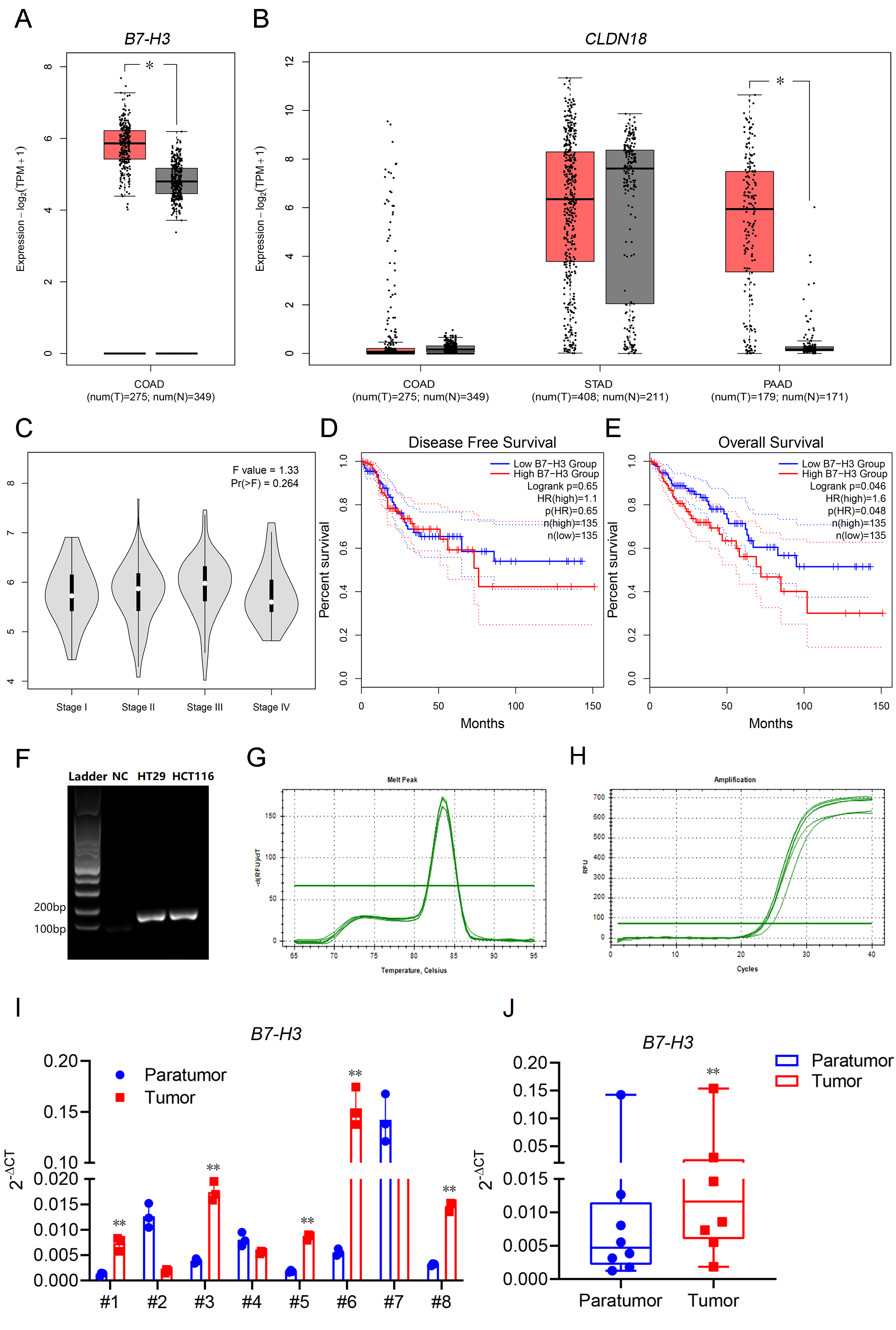
**

**Figure S1. The expression of B7-H3, rather than Claudin18.2, is significantly upregulated in CRC**

**A**. Higher transcripts of B7-H3 in CRC tumor than paired normal tissues. Data are presented as mean ± SD. * *p* < 0.05. COAD: colon adenocarcinoma

**B**. Claudin18.2 showed minimal expression in CRC but high expression in STAD and PAAD samples. Data are presented as mean ± SD. * *p* < 0.05. STAD: stomach adenocarcinoma; PAAD: pancreatic adenocarcinoma

**C**. The expression of B7-H3 showed no significant difference among different stages of CRC.

**D-E**. Relationship between the expression of B7-H3 and disease-free survival (D) or overall survival (E). Data was analyzed by Kaplan-Meier survival analysis and log-rank test. A-E. all the data from TCGA.

**F**. Electrophoresis figure of PCR product amplified with B7-H3 specific primers using HT29 and HCT116 cells. NC, negative control.

**G**. Melt curve of B7-H3 PCR product using HT29 and HCT116 cells.

**H**. Amplification curve of quantitative PCR amplified with B7-H3 specific primers.

**I-J**. B7-H3 mRNA levels were assayed in tumor and paratumor tissues from eight patients (I), revealing a significantly higher expression in tumor tissues compared to paratumor tissues (J). Data are presented as mean ± SD. ** *p* < 0.01

**
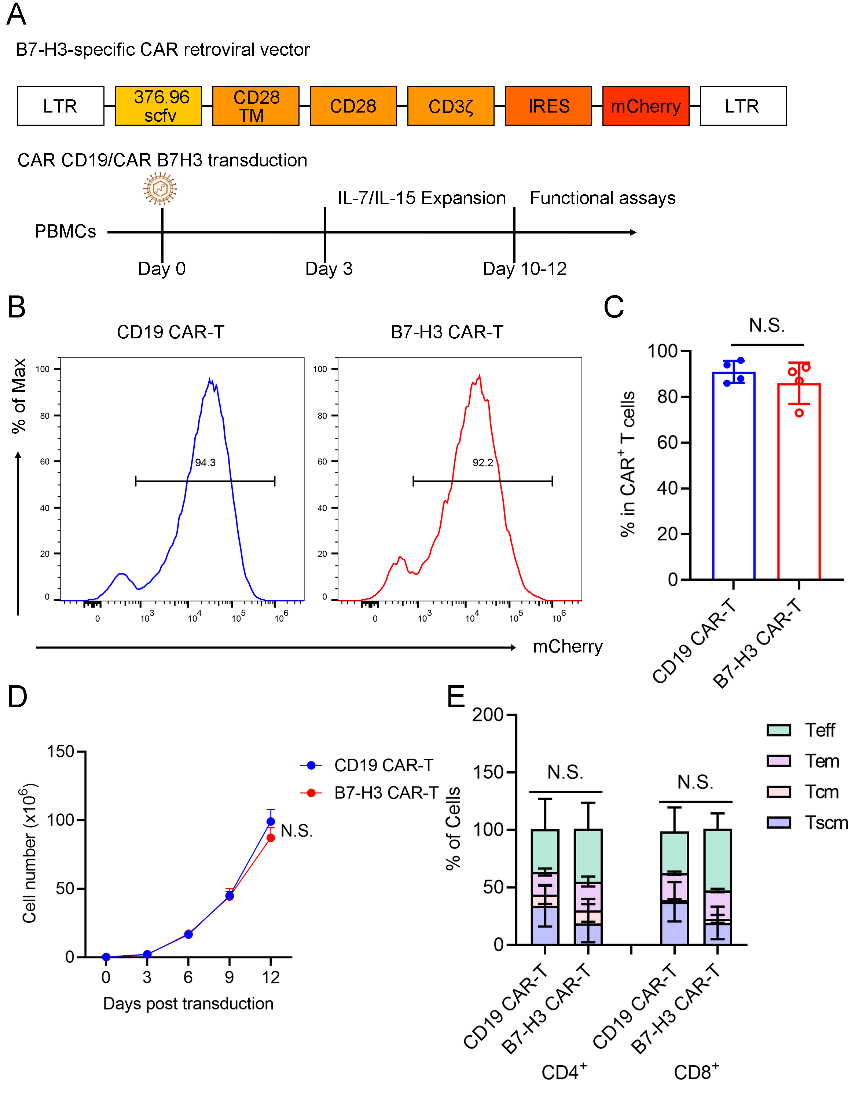
**

**Figure S2. Construction of CAR-T cells targeting B7-H3**

**A**. Schematic diagram illustrating the vector encoding B7-H3 CAR and the productive process of generating B7-H3 CAR-T cells.

**B.** Representative expression of CD19 or B7-H3 CAR-T cells in transduced human T cells.

**C.** Summary of the CD19 and B7-H3 CAR-T cells transduction efficiency. Data are presented as mean ± SD. N.S., no significance.

**D.** Expansion kinetics of CD19 and B7-H3 CAR-T cells in vitro. Data are presented as mean ± SD. N.S., no significance.

**E.** Phenotypic analysis of CAR-T cells at 12 days post transduction showing the frequency of effector T cells (Teff, CD45RA^+^CCR7^-^), effector memory T cells (Tem, CD45RA^-^CCR7^-^), central memory T cells (Tcm, CD45RA^-^CCR7^+^) and stem cell memory T cells (Tscm, CD45RA^+^CCR7^+^) in CD4^+^ T cells and in CD8^+^ T cells. Data are presented as mean ± SD. N.S., no significance.


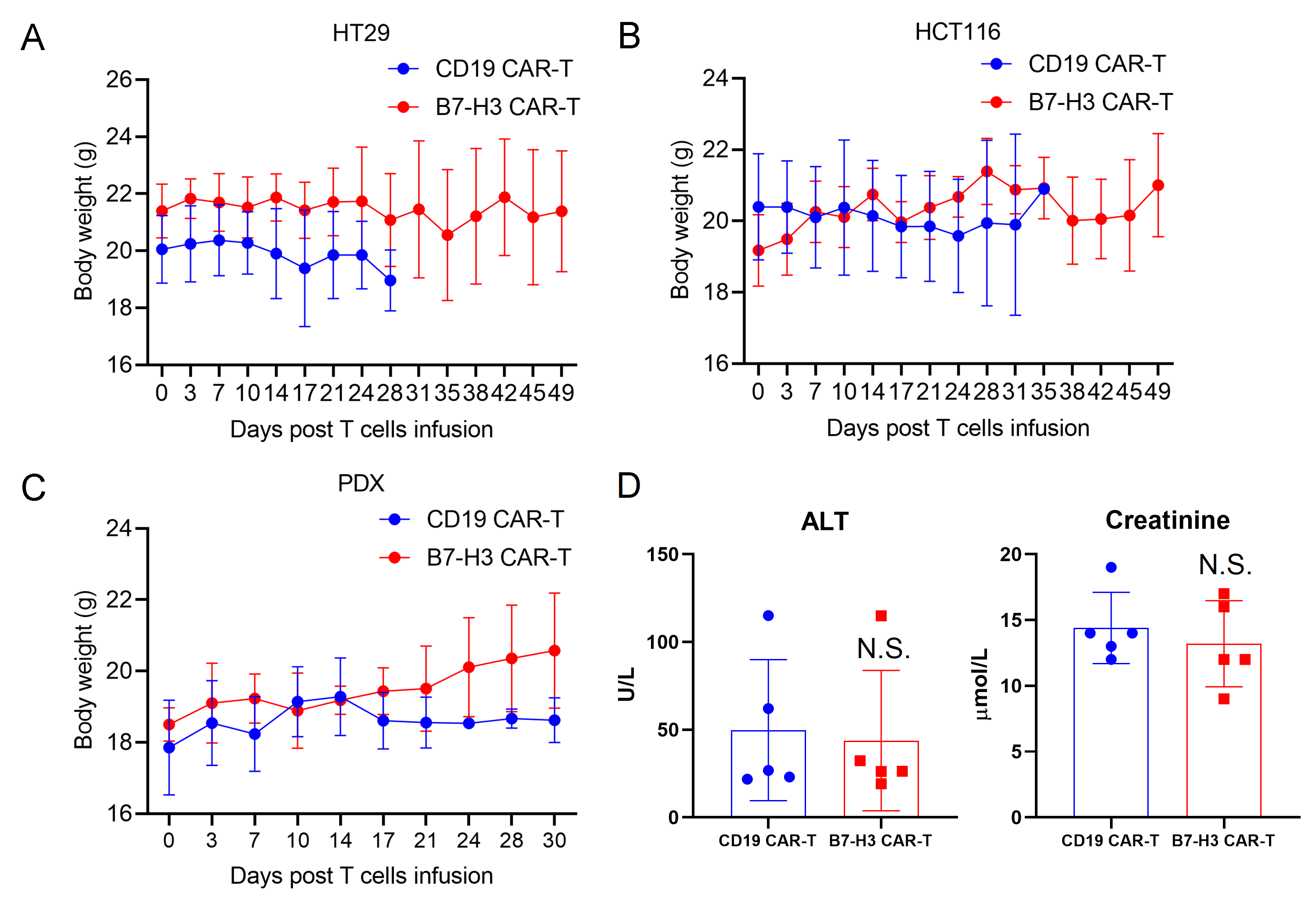


**Figure S3. Body weights of mice receiving different treatments**

**A-C**. Changes of body weight of mice bearing xenografts with HT29 (A), HCT116 (B) and PDX liver metastases (C) after treatment with CD19 or B7-H3 CAR-T cells. Data are presented as mean ± SD.

**D**.  Alanine transaminase (ALT) and creatinine of the mice after CAR-T cell therapy (30 days) were measured on automatic biochemical analyser. Data are presented as mean ± SD. N.S., no significance.

**
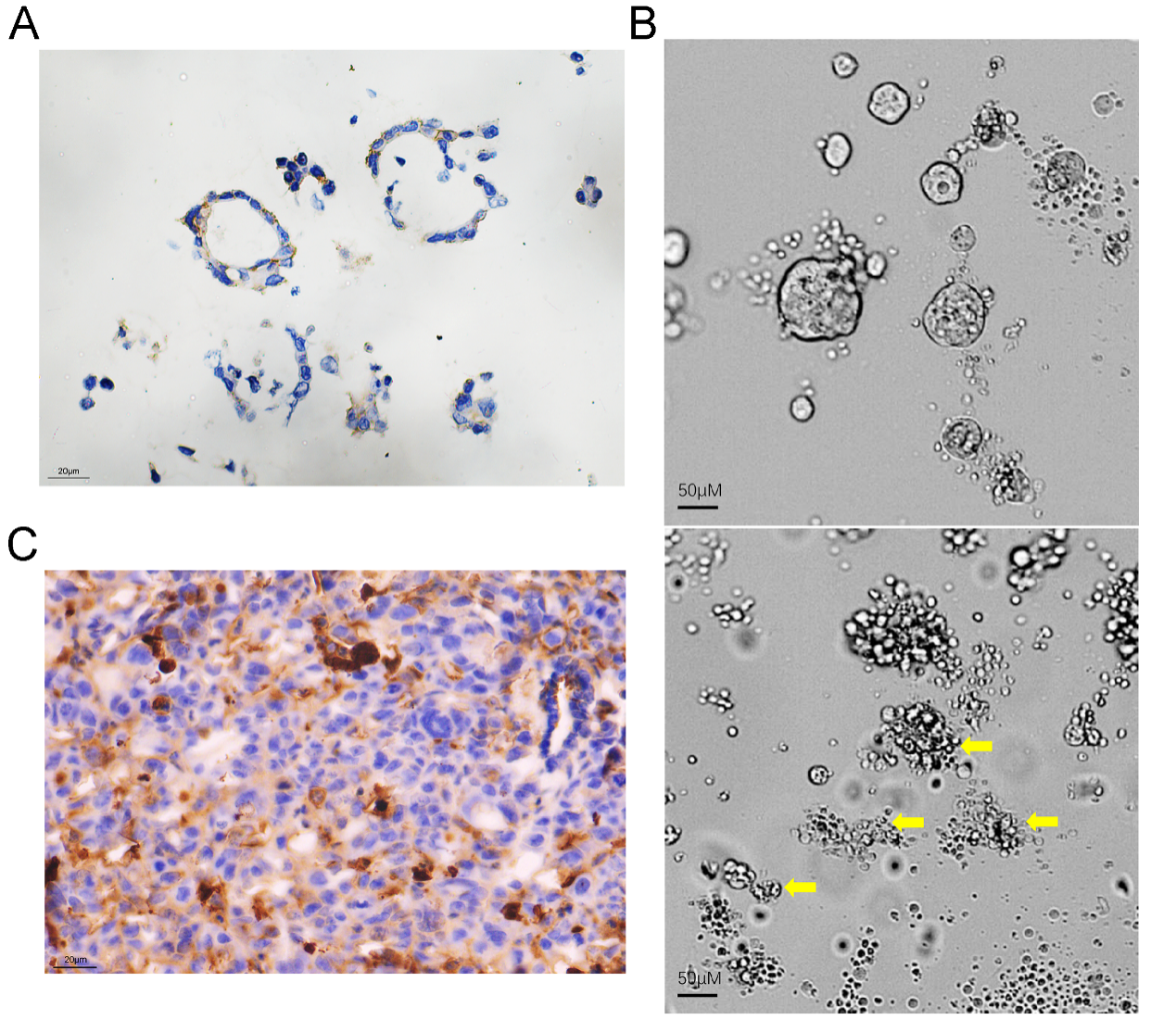
**

**Figure S4. IHC staining of B7-H3**

**A**. Representative image from IHC staining of B7-H3 in CRC organoids. Scale bar = 20 μm.

**B**. Morphology of CRC organoids co-cultured with CD19 (upper) or B7-H3 CAR-T cells (bottom). Arrows show the organoids following B7-H3 CAR-T cells attacking. Scale bar = 50 μm.

**C**. Representative IHC staining image of B7-H3 in PDX tumor tissue. Scale bar = 20 μm.


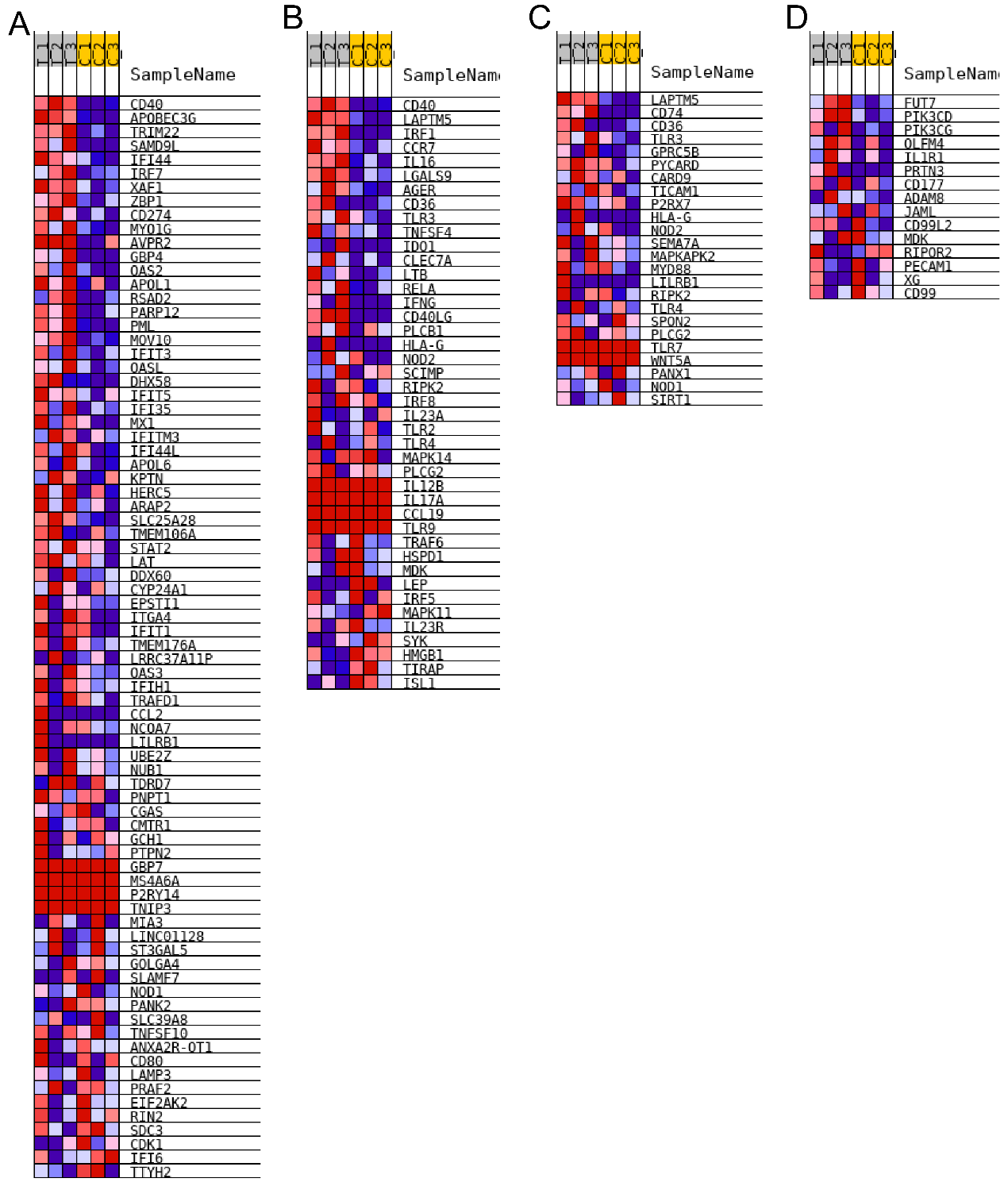


**Figure S5. Genes involved in the signal pathways presented in Figure 5D**

**A**. Genes involved in the pathway of Interferon Induced Antiviral Module.

**B**. Genes involved in the pathway of Interleukin 12 Production.

**C**. Genes involved in the pathway of Positive Regulation of Macrophage Cytokine Production.

**D**. Genes involved in the pathway of Neutrophil Extravasation.
